# King's College Hospital

**Published:** 1911-03-04

**Authors:** W. F. D. Smith

**Affiliations:** 3 Grosvenor Place, W.; 3 Grosvenor Place, W.


### INSTITUTIONAL LETTER-BOX.

### KING'S COLLEGE HOSPITAL.

*To the Editor of* THE HOSPITAL.

SIR,—We venture once more to ask you to give publicity to the needs of the King's College Hospital Removal Fund. Seven years ago, when the decision was taken to remove the Hospital to South London, the appeal then made was generously responded to, and in each succeeding year additions to the Fund have been made, bringing the total sum to £190,000, excluding the value of the site.

In the summer of 1909 his Majesty King Edward laid the foundation stone; since that date building has rapidly proceeded, and the funds at the disposal of the committee will soon be exhausted. The out-patient and casualty department and bathing establishment, together with the administration block, are now roofed in and nearly completed; it remains to build the chapel, ward blocks, operation theatres, and some other smaller departments.

For these the Committee ask for a further sum of £150,000 with which to bring the building to a stage when it can open its doors to the many poor people of South London who are so much in need of hospital accommodation. We earnestly hope that this happy event may not be delayed by lack of funds, and it is for this reason that we venture once more to appeal to the public.

Donations may be sent to the Treasurer, Removal Fund, King's College Hospital, W.C., and will be added to the amount to be announced at the dinner to be held on behalf of the appeal at the Connaught Rooms on May 4.—Yours faithfully,

3 Grosvenor Place, W., February 25.

